# Methylphenidate and Short-Term Cardiovascular Risk

**DOI:** 10.1001/jamanetworkopen.2024.1349

**Published:** 2024-03-06

**Authors:** Miguel Garcia-Argibay, Paul-Christian Bürkner, Paul Lichtenstein, Le Zhang, Brian M. D’Onofrio, Pontus Andell, Zheng Chang, Samuele Cortese, Henrik Larsson

**Affiliations:** 1 School of Medical Sciences, Örebro University, Faculty of Medicine and Health, Örebro, Sweden; 2 Department of Medical Epidemiology and Biostatistics, Karolinska Institutet, Stockholm, Sweden; 3 Cluster of Excellence SimTech, University of Stuttgart, Stuttgart, Germany; 4 The Department of Psychological and Brain Sciences at Indiana University, Bloomington; 5 Unit of Cardiology, Department of Medicine, Karolinska Institutet, Stockholm, Sweden; 6 Heart and Vascular Division, Karolinska University Hospital, Stockholm, Sweden; 7 School of Psychology, University of Southampton, Southampton, United Kingdom; 8 Clinical and Experimental Sciences (CNS and Psychiatry), Faculty of Medicine, University of Southampton, Southampton, United Kingdom; 9 Solent NHS Trust, Southampton, United Kingdom; 10 Hassenfeld Children’s Hospital at NYU Langone, New York University Child Study Center, New York City; 11 DiMePRe-J-Department of Precision and Regenerative Medicine-Jonic Area, University of Bari Aldo Moro, Bari, Italy

## Abstract

**Importance:**

There are concerns about the safety of medications for treatment of attention-deficit/hyperactivity disorder (ADHD), with mixed evidence on possible cardiovascular risk.

**Objective:**

To assess whether short-term methylphenidate use is associated with risk of cardiovascular events.

**Design, Setting, and Participants:**

This retrospective, population-based cohort study was based on national Swedish registry data. Participants were individuals with ADHD aged 12 to 60 years with dispensed prescriptions of methylphenidate between January 1, 2007, and June 30, 2012. Each person receiving methylphenidate (n = 26 710) was matched on birth date, sex, and county to up to 10 nonusers without ADHD (n = 225 672). Statistical analyses were performed from September 13, 2022, to May 16, 2023.

**Main Outcomes and Measures:**

Rates of cardiovascular events, including ischemic heart disease, venous thromboembolism, heart failure, or tachyarrhythmias, 1 year before methylphenidate treatment and 6 months after treatment initiation were compared between individuals receiving methylphenidate and matched controls using a bayesian within-individual design. Analyses were stratified by history of cardiovascular events.

**Results:**

The cohort included 252 382 individuals (15 442 [57.8% men]; median age, 20 (IQR, 15-31) years). The overall incidence of cardiovascular events was 1.51 per 10 000 person-weeks (95% highest density interval [HDI], 1.35-1.69) for individuals receiving methylphenidate and 0.77 (95% HDI, 0.73-0.82) for the matched controls. Individuals treated with methylphenidate had an 87% posterior probability of having a higher rate of cardiovascular events after treatment initiation (incidence rate ratio [IRR], 1.41; 95% HDI, 1.09-1.88) compared with matched controls (IRR, 1.18; 95% HDI, 1.02-1.37). The posterior probabilities were 70% for at least a 10% increased risk of cardiovascular events in individuals receiving methylphenidate vs 49% in matched controls. No difference was found in this risk between individuals with and without a history of cardiovascular disease (IRR, 1.11; 95% HDI, 0.58-2.13).

**Conclusions and Relevance:**

In this cohort study, individuals receiving methylphenidate had a small increased cardiovascular risk vs matched controls in the 6 months after treatment initiation. However, there was little evidence for an increased risk of 20% or higher and for differences in risk increase between people with and without a history of cardiovascular disease. Therefore, before treatment initiation, careful consideration of the risk-benefit trade-off of methylphenidate would be useful, regardless of cardiovascular history.

## Key Points

**Question:**

What is the cardiovascular risk associated with short-term methylphenidate use?

**Findings:**

In this cohort study of 252 382 individuals, methylphenidate-treated individuals showed a higher (10%) cardiovascular event rate compared with non-treated matched controls in the 6 months after treatment. However, there was no evidence for a more substantial difference (≥20%) or difference based on preexisting cardiovascular disease.

**Meaning:**

The findings of this study suggest that the small cardiovascular risk associated with short-term methylphenidate use should not be a reason to withhold treatment but suggest the need for individualized risk-benefit assessment and risk monitoring.

## Teaser

This cohort study examines the risks of cardiovascular disease after 6 months of methylphenidate treatment in individuals with attention-deficit/hyperactivity disorder.

## Introduction

Attention-deficit/hyperactivity disorder (ADHD) is a common neurodevelopmental condition affecting approximately 5% to 10% of school-aged children and 2% to 5% of adults.^1^ Characterized by persistent and developmentally inappropriate inattention and/or hyperactivity/impulsivity, ADHD can co-occur with several mental and physical conditions,^2,3,4,5,6,7^ including cardiovascular disease (CVD^4^), leading to a substantial burden on the health care system.^8,9^

Methylphenidate, a central nervous system stimulant, has long been used as a first-line medication for the treatment of ADHD. It has been shown to be efficacious in randomized clinical trials and useful in observational studies in decreasing ADHD symptom severity and related impairments.^10^ However, despite its widespread use, there is still ongoing debate about the safety of methylphenidate. Among possible adverse effects, there is evidence showing small but statistically significant increases in blood pressure and heart rate following its use.^11,12^ However, it is not clear whether and to what extent this increase in blood pressure is associated with an increased risk of cardiovascular conditions, as existing evidence is mixed. Some prospective and retrospective cohort studies have reported an increased risk of cardiovascular events in individuals receiving methylphenidate treatment.^13,14,15,16,17,18^ In contrast, other retrospective, population-based cohort studies did not find any association between methylphenidate use and cardiovascular events.^19,20,21,22,23,24^

There are 2 crucial limitations in previous studies investigating the possible association between methylphenidate use and cardiovascular events. First, several studies did not adequately control unmeasured confounding factors that may lead to biased estimations.^25,26,27,28,29,30^ A within-individual design, in which each individual serves as their own control, can address, at least partially, this methodologic limitation. Second, available studies based their conclusions on null hypothesis significance testing. While this is a widely used statistical method, it has several limitations.^25,26^ Bayesian inference offers several advantages over null hypothesis significance testing,^27,28,29^ and is often considered to be a more reliable and flexible method for statistical analysis.^30^ Bayesian statistics, with its inherent capacity to express parameters and hypotheses as probabilities, offers clinicians an analytical framework with potential utility within the realm of medical practice. By encapsulating uncertainty through the lens of probabilities, bayesian inference allows for a more comprehensive and nuanced understanding of clinical phenomena, facilitating informed decision-making and fostering personalized patient care.

We aimed to conduct what is, to our knowledge, the first bayesian population-based self-controlled cohort study assessing the risk of cardiovascular adverse events 6 months after the start of methylphenidate treatment in individuals with ADHD with and without a history of CVD.

## Methods

The study was approved by the regional ethical review board in Stockholm, Sweden. The need for informed consent was waived because the study was register-based and data on the included individuals were deidentified. We followed the Strengthening the Reporting of Observational Studies in Epidemiology (STROBE) reporting guideline.^31^

### Study Design and Population

This study was based on the linkage of 3 population-based registers in Sweden via unique personal identity numbers^32^: the Total Population Register, the Prescribed Drug Register (PDR), and the National Patient Register (NPR). The Total Population Register includes data on birth, county, sex, death, and migration date. The PDR contains records of all prescribed drugs dispensed to the entire population of Sweden since 2005, with more than 99% national coverage. The NPR includes diagnosis codes and dates of both somatic and psychiatric inpatient care and outpatient specialist visits according to the *International Statistical Classification of Diseases and Related Health Problems, Tenth Revision* (ICD-10).

In this population-based study, we used a multilevel design—time nested within individuals—to compare the incidence of cardiovascular events within the same person (ie, each person served as their own control) between 2 periods: 12 months before (baseline) and 6 months after methylphenidate initiation (follow-up). By extending the baseline period to 12 months, we aimed to strengthen the precision of the estimated pretreatment event rate for each individual. This provides a more reliable basis for comparison when assessing the posttreatment change in event rate.

Furthermore, we compared the incidence of cardiovascular events in individuals with ADHD treated with methylphenidate and in controls with no history of ADHD or ADHD medication use. Individuals receiving methylphenidate were individually exact matched without replacement by birth date (month and year), sex, and county to up to 10 population-based controls. In matched controls, the date of methylphenidate initiation was set to be the same as their methylphenidate counterparts.

The study population included individuals with new use of methylphenidate aged between 12 and 60 years at the start of methylphenidate treatment. Individuals receiving methylphenidate were defined, in accordance with previous research,^33^ as those who had a first dispensed methylphenidate prescription (Anatomical Therapeutic Chemical code N06BA04) between January 1, 2007, and June 30, 2012, as identified in the PDR. This definition includes those who had started methylphenidate treatment for the first time and those without any recorded prescription of methylphenidate for at least 1.5 years since the beginning of the PDR (July 1, 2005). Individuals were excluded from the study if they (1) received ADHD medications other than methylphenidate during the study period or did not have a diagnosis of ADHD in the NPR, (2) immigrated to Sweden after the start of period 1 (ie, 1 year before treatment initiation), or (3) died or emigrated before the end of period 2 (ie, 6 months after treatment initiation).

### Outcomes

Potential cardiovascular events were defined as those leading to any inpatient admission or outpatient attendance for ischemic heart disease (acute myocardial infarction or acute coronary syndrome), cerebrovascular disease (subarachnoid bleeding, hemorrhagic stroke, ischemic stroke, or other cerebrovascular disease), venous thromboembolism (deep vein thrombosis or pulmonary emboli), heart failure, or tachyarrhythmias (atrial fibrillation/flutter, supraventricular tachycardia, ventricular tachycardia, or cardiac arrest) until the end of the study period (December 31, 2013). eTable 1 in Supplement 1 reports all *ICD-10* codes used for each diagnosis. Patients with at least 1 of the aforementioned diagnoses before the start of the follow-up period (ie, 1 year before treatment initiation) were considered to have a history of CVD.

### Statistical Analysis

A bayesian multilevel negative binomial model with a random intercept for each individual was fitted to compare the incidence of cardiovascular events 1 year before methylphenidate initiation and after 6 months of methylphenidate treatment. In consecutive joint analyses, an interaction term was included between follow-up and the methylphenidate group vs the control group to assess whether there was any difference in the incidence of cardiovascular events between individuals receiving methylphenidate and matched controls in the follow-up compared with baseline. Analyses were further performed separately in persons with and without a history of CVD who were compared by including a 3-way interaction. All models were adjusted for mean-centered age at the start of methylphenidate treatment (confounder), and the log of person-weeks of baseline and follow-up were used as offset. Model parameters were estimated with the No-U-Turn Hamiltonian Monte Carlo sampling in Stan^34^ (target average acceptance probability set to 0.97) using the r-package brms.^35^ Additional information on priors, convergence assessment, and model fit can be found in the eMethods in Supplement 1.

We estimated the posterior probabilities that the difference in the incidence rate for cardiovascular events between baseline and follow-up for individuals receiving methylphenidate and matched controls (and the difference between them) exceeded a range of potential values (incidence rate ratio [IRR] >1.0, 1.1, 1.2, 1.3, and 1.4). Only individuals with at least 1 cardiovascular event during the selected 2 periods contributed to the analysis. Results are presented as IRR and median log rate with 95% highest density intervals (HDIs), and the region of practical equivalence (89% HDI region of practical equivalence [ROPE]: range ±0.1).^30^ Data preparation was performed using SAS software, version 9.4 (SAS Institute Inc), and analyses were conducted using R, version 4.2.3.^36^ Statistical analyses were performed from September 13, 2022, to May 16, 2023.

## Results

We identified 34 457 individuals who started methylphenidate treatment between January 1, 2007, and June 30, 2012. Among those, 369 individuals (1%) were excluded because of death (0.2%) and emigration or immigration (0.8%) during the study period, and 7378 (21.6%) were excluded due to not fulfilling the age criterion. Among 296 644 matched controls, 22 537 (7.6%) were excluded because of death (0.3%) and emigration or immigration during the study period (7.3%), and 48 435 (16.3%) due to age restrictions. A total of 252 382 individuals were included for analysis. Overall age was 20 (IQR, 15-31) years. The methylphenidate cohort included 26 710 individuals with a median age of 22 years (IQR, 16-34), including 11 268 (42.2%) females and 15 442 (57.8%) males. Among those receiving methylphenidate treatment, a history of cardiovascular events was reported in 383 (1.4%) individuals, and 187 (0.7%) participants had at least 1 cardiovascular event during the study period (Table 1). Among the matched controls, 1338 (0.6%) had a history of cardiovascular events and 631 (0.3%) had at least 1 cardiovascular event during the study period. The overall incidence of cardiovascular events was 1.51 per 10 000 person-weeks (95% HDI, 1.35-1.69) for the methylphenidate group and 0.77 (95% HDI, 0.73-0.82) for the matched controls. Among those with a history of CVD, the incidence was 42.18 (95% HDI, 35.16-50.08) per 10 000 person-weeks and, in those without a history of CVD, 0.92 (95% HDI, 0.80-1.06) events per 10 000 person-weeks. In the matched controls, the overall incidence was 56.54 (95% HDI, 52.05-61.24) in those with a history of CVD and, in those without a history of CVD, 0.44 (95% HDI, 0.41-0.47).

**Table 1.** Characteristics of the Study Population

Variable	Group, No. (%)	
	Methylphenidate users (n = 26 710)	Matched controls (n = 225 672)
Sex		
Female	11 268 (42.2)	94 317 (41.8)
Male	15 442 (57.8)	131 355 (58.2)
History of CVD		
No	26 327 (98.6)	224 334 (99.4)
Yes	383 (1.4)	1338 (0.6)
Age at treatment initiation, median (IQR), y	22 (16-34)	20 (15-30)
Individuals with at least 1 cardiovascular event		
Baseline	110 (0.4)	395 (0.2)
Follow-up	77 (0.3)	273 (0.1)
No. of cardiovascular events (person-weeks)	308 (2 033 776)	1331 (17 183 311)
With history of CVD	123 (29 163)	576 (101 879)
Without history of CVD	185 (2 004 613)	755 (17 081 432)
Incidence rate (95% CI) per 10 000 person-weeks^a^		
Baseline	1.34 (1.16-1.55)	0.89 (0.84-0.95)
Follow-up	1.89 (1.58-2.26)	0.89 (0.81-0.97)

Abbreviation: CVD, cardiovascular disease.

^a^ Baseline, 1 year before treatment initiation; follow-up, 6-month period after treatment initiation.

**Figure 1.**
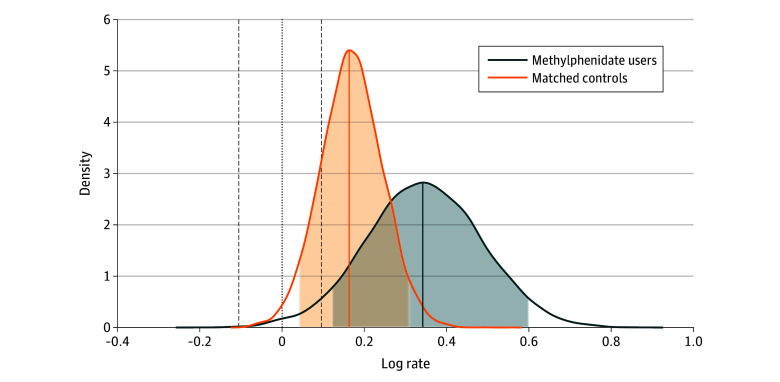
Posterior Distribution of the Log Rate for Methylphenidate Users and Matched Controls Dashed lines represent the upper and lower bounds for the region of practical equivalence. Shaded areas contain the 95% highest density interval.

**Figure 2.**
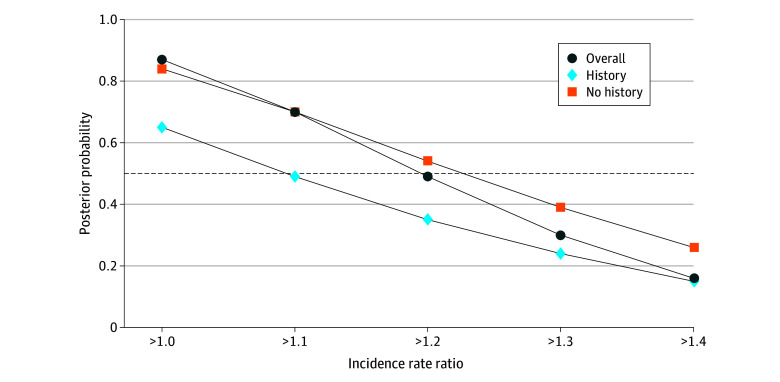
Posterior Probabilities for Incidence Rate Ratio Between Methylphenidate Users and Matched Controls Using Different Thresholds The dashed line indicates posterior probability of 0.5.

Model fit and convergence information can be found in the eResults, eTable 2, eFigure 1, and eFigure 2 in Supplement 1. When comparing the incidence rate of cardiovascular events 1 year before methylphenidate initiation with 6 months after treatment (ie, baseline vs follow-up), we found an increased rate (IRR, 1.41; 95% HDI, 1.09-1.88; 0% in ROPE) with a 99% posterior probability for any difference in the rate between baseline and follow-up, 96% posterior probability for a difference larger than 10%, and 87% posterior probability for a difference larger than 20%. For the matched controls, we found an increased rate of cardiovascular events (IRR, 1.18; 95% HDI, 1.02-1.37; 14% in ROPE) with a 98% posterior probability for any difference, an 82% posterior probability for a difference larger than 10% (Figure 1), and a 40% probability for a difference larger than 20%. When comparing individuals receiving methylphenidate with matched controls, we found an 87% posterior probability for any difference in the IRR of cardiovascular events (IRR, 1.19; 95% HDI, 0.88-1.64), a 70% posterior probability for a difference larger than 10%, and a 49% posterior probability for a difference larger than 20% (Table 2). Figure 2 summarizes all posterior probabilities at different thresholds. The posterior probabilities decreased substantially for all risk differences equal to or greater than 20%.

**Table 2.** Results From the Multilevel Negative Binomial Regression^a^

Variable and model	Median log rate	% in ROPE	IRR (95% HDI)	Posterior probability of IRR >specified threshold, %				
				1.0	1.1	1.2	1.3	1.4
Overall								
Methylphenidate: baseline vs follow-up	0.34	0	1.41 (1.09-1.88)	99	96	87	72	52
Controls, baseline vs follow-up	0.17	13.76	1.18 (1.02-1.37)	98	82	40	10	1
Methylphenidate vs controls	0.18	28.65	1.19 (0.88-1.64)	87	70	49	30	16
No previous CVD								
Methylphenidate: baseline vs follow-up	0.55	0	1.74 (1.24-2.42)	100	99	98	95	88
Controls: baseline vs follow-up	0.35	0	1.42 (1.15-1.72)	100	100	95	81	55
Methylphenidate vs controls	0.20	25.92	1.23 (0.84-1.87)	84	70	54	39	26
Previous CVD								
Methylphenidate: baseline vs follow-up	0.01	39	1.00 (0.65-1.54)	48	31	19	10	5
Controls: baseline vs follow-up	–0.10	50.17	0.90 (0.75-1.11)	17	3	0	0	0
Methylphenidate vs controls	0.09	33.77	1.09 (0.68-1.74)	65	49	35	24	15
Difference								
Previous CVD vs no history	0.11	24.90	1.11 (0.58-2.13)	62	50	36	23	11

Abbreviations: CVD, cardiovascular disease; HDI, highest density interval; IRR, incidence rate ratio; ROPE, region of practical equivalence.

^a^ Baseline was used as the reference category.

In individuals without a history of CVD, we found a 70% posterior probability for a 10% difference in the rate of cardiovascular events between individuals receiving methylphenidate and matched controls (IRR, 1.23; 95% HDI, 0.84-1.87). In individuals with a history of CVD, we found a 65% posterior probability for any difference in the IRR of cardiovascular events between individuals receiving methylphenidate and matched controls (IRR, 1.09; 95% HDI, 0.68-1.74), a 49% posterior probability for differences larger than 10%, and a 35% probability for differences larger than 20%. When comparing those with and without a history of CVD, we found a 62% probability that individuals receiving methylphenidate treatment without a history of CVD had a higher risk of cardiovascular events compared with those with a history (IRR, 1.11; 95% HDI, 0.58-2.13).

## Discussion

In this population-based cohort study of more than 250 000 children, adolescents, and adults, including 26 710 individuals receiving methylphenidate, we found evidence (87% posterior probability) of an increased rate of cardiovascular events associated with methylphenidate use after the first 6 months of pharmacologic treatment compared with matched controls, with inconclusive evidence regarding differences between those with vs without a history of CVD. When considering at least a 10% increased rate of cardiovascular events, the probability of finding a difference between individuals receiving methylphenidate and controls was lower (70%), and the probability decreased even further for larger differences in the rates of cardiovascular events.

In contrast with recent meta-analytic evidence from observational studies^24^ showing no association between ADHD medication use and increased risk of CVD (relative risk, 1.22; 95% CI, 0.88-1.68), we found an increased cardiovascular risk with methylphenidate use. However, when quantifying this risk, we found little evidence for an increased risk of 20% or higher. We also found insufficient evidence for a difference in risk between individuals with and without a history of CVD. Our findings align with a Danish register-based study^17^ reporting an elevated risk of CVD in individuals who started pharmacologic treatment for ADHD, as well as a lack of evidence supporting a difference in CVD risk between individuals with and without preexisting CVD. Furthermore, the increased IRR 1 year before the start of methylphenidate in the ADHD group aligns with recent studies suggesting that ADHD itself confers significantly greater cardiovascular risk.^4^

The somewhat higher risk in those without a history of CVD may be accounted for by the fact that individuals with a history of CVD are more closely monitored for cardiovascular risk factors and receive more intensive treatment to manage those risk factors. For instance, individuals with a history of CVD may be more likely to receive medications to manage hypertension, high low-density lipoprotein cholesterol levels, or diabetes, which are all risk factors for CVD. They may also be more likely to receive regular medical check-ups and undergo more frequent testing to monitor their CVD risk, ultimately reducing their recurrent event risk. In contrast, individuals without a history of CVD who are prescribed methylphenidate for ADHD may not receive the same level of monitoring and treatment for CVD risk factors. Therefore, they may be more vulnerable to the potential cardiovascular effects of methylphenidate use.

While the precise mechanisms by which methylphenidate could increase the risk of cardiovascular events remain elusive, it has been proposed that catecholamines may play an important role. Methylphenidate has been shown to increase catecholamine neurotransmission,^37^ which in turn can activate cardiovascular β_1_ adrenoceptors^38^ that might predispose to a range of cardiovascular events, including arrhythmias, myocardial infarction, and cardiac death. Furthermore, the sympathomimetic actions of methylphenidate could result in an increase in circulating norepinephrine levels, with attendant effects on peripheral vascular tone and vasoconstriction. Additionally, an increase in circulating catecholamine levels has been associated with a decrease in cardiac baroreflex sensitivity,^39^ which could negatively affect the heart rate response to stress. The present study was not designed to investigate the mechanisms underlying the observed association between methylphenidate and cardiovascular events. Future studies need to replicate our results, ideally using approaches that avoid null hypothesis significance testing and control for confounding, to determine the safety cardiovascular profile of methylphenidate and identify the underlying mechanisms.

### Strengths and Limitations

Strengths of this study include the use of the PDR that allowed us to identify and include a large number of individuals receiving methylphenidate, with almost complete coverage (>99%) of dispensed prescription drugs in Sweden. Moreover, with the inclusion of diagnoses based on inpatient and outpatient medical records, we avoided faulty recollection of cardiovascular events. Furthermore, by using a within-individual approach, we compared the rate of cardiovascular events before and after methylphenidate treatment within the same individual (ie, each individual was their own control) adjusting for unmeasured constant factors. The variance of the individual-level residual addressed the influence of both measured and unmeasured sources. Additionally, to account for the possible effect of age rather than methylphenidate treatment, individuals with ADHD were matched by sex, birth date, and county, and age was included at the start of methylphenidate treatment as a between-level variable. As age was not a significant between-level predictor, it is unlikely that we introduced bias by using a 1-year baseline period.

Our findings should be interpreted considering some limitations. First, our data are observational rather than experimental, and thus causality cannot be inferred. Second, due to the lack of information on methylphenidate dose, we were unable to assess whether the cardiovascular risk varied as a function of the dose (eg, higher doses may be associated with an increased rate of cardiovascular events). Third, as with all register-based studies, we had no information on adherence. The number of days that a given filled prescription was supplied for does not necessarily represent the actual time of use, so that we could not rule out nonadherence. By using an intention-to-treat approach (ie, we assumed individuals used methylphenidate as prescribed), we might have potentially underestimated the associations. Fourth, as individuals older than 60 years were not included in this study, our conclusions derived from a restricted sample might not be extrapolated to broader population. Fifth, given that the Swedish PDR started in 2005, there were no records of prescriptions for ADHD before this date. As such, we only included individuals with new use of methylphenidate or those without any recorded prescription of methylphenidate for at least 1.5 years since the beginning of the PDR. It is possible that some individuals underwent and stopped methylphenidate treatment before 2005, exacerbating the susceptibility of cardiovascular events. This could potentially have inflated the association, assuming that methylphenidate is linked to an increased number of cardiovascular events.

There are several limitations to consider when generalizing the results of the current research findings on the association between methylphenidate and CVD to other countries, particularly those with different medical practices, national health care systems, and cultural factors than Sweden. The findings of this study were based on data collected from a Swedish population, which may not be representative of other populations with varying demographic characteristics, lifestyles, and health behaviors. Medical practice and management of cardiovascular risk factors (eg, hypertension and hyperlipidemia) may also differ across countries, which could impact the generalizability of the results. Additionally, national health care systems, including access to health care services, prescription patterns, and drug regulations, can vary substantially between countries, potentially affecting the use and safety of methylphenidate. Moreover, cultural factors, such as attitudes toward medication use, patient preferences, and societal norms related to cardiovascular health and stimulant medications, may also vary across countries and influence the generalizability of the findings. Therefore, caution should be exercised when extrapolating the results of this study to other countries, and further research in diverse populations is warranted to better understand the generalizability of the observed association between methylphenidate and cardiovascular disease. In addition, although CVD misclassification cannot be completely ruled out, it is highly unlikely due to the nature and severity of CVD and the high positive predictive value from the NPR.^40^

## Conclusions

This cohort study found evidence supporting a small (10%) increased risk of cardiovascular events in individuals receiving methylphenidate compared with matched controls after 6 months of treatment. The probability of finding a difference in risk between users and nonusers decreased when considering risk of 20% or larger, with no evidence of differences between those with and without a CVD history. Overall, while the findings suggest the decision to initiate methylphenidate should incorporate considerations of potential adverse cardiovascular effects, they do not establish evidence of markedly elevated cardiovascular risk. Rather, they provide probabilistic evidence most consistent with a small increased rate of cardiovascular events attributable to methylphenidate exposure over 6 months that warrants consideration among the broader benefits and risks of treatment for individual patients.

## Notes

Supplement 1. **eMethods.** Bayesian Statistics and Detailed Methods **eResults.** Detailed Results **eTable 1.** Diagnoses and *ICD-10* Codes to Define the Occurrence of Short-Term Cardiovascular Events **eTable 2.** Model Fit Comparison Among Negative Binomial, Poisson, Zero-Inflated Poisson, and Zero-Inflated Negative Binomial **eFigure 1.** Trace Plot of the Four MCMC Chains After Burn-In **eFigure 2.** Graphical Posterior Predictive Checking Using an Overlay of Density Plots and Distribution of Mean Values in MPH Users and Matched Controls Based on 300 Posterior Draws
eReferences

Supplement 2.
Data Sharing Statement
