# Supplementary material for: Methylphenidate and Short-Term Cardiovascular Risk
Source: JAMA Netw Open. 2024 Mar 6;7(3):e241349. doi: 10.1001/jamanetworkopen.2024.1349 (PMC10918505; doi:10.1001/jamanetworkopen.2024.1349)
Supplement: Supplement 1. — eMethods. Bayesian Statistics and Detailed Methods eResults. Detailed Results eTable 1. Diagnoses and ICD-10 Codes to Define the Occurrence of Short-Term Cardiovascular Events eTable 2. Model Fit Comparison Among Negative Binomial, Poisson, Zero-Inflated Poisson, and Zero-Inflated Negative Binomial eFigure 1. Trace Plot of the Four MCMC Chains After Burn-In eFigure 2. Graphical Posterior Predictive Checking Using an Overlay of Density Plots and Distribution of Mean Values in MPH Users and Matched Controls Based on 300 Posterior Draws eReferences [file jamanetwopen-e241349-s001.pdf]

Supplementary Online Content

Garcia-Argibay M, Bürkner PC, Lichtenstein P, et al. Methylphenidate and short-term cardiovascular risk. *JAMA Netw Open*. 2024;7(3):e241349. doi:10.1001/jamanetworkopen.2024.1349

eIntroduction. Bayesian Statistics

eMethods. Detailed Methods

eResults. Detailed Results

eTable 1. Diagnoses and *ICD-10* Codes to Define the Occurrence of Short-Term Cardiovascular Events

eTable 2. Model Fit Comparison Among Negative Binomial, Poisson, Zero-Inflated Poisson, and Zero-Inflated Negative Binomial

eFigure 1. Trace Plot of the Four MCMC Chains After Burn-In

eFigure 2. Graphical Posterior Predictive Checking Using an Overlay of Density Plots and Distribution of Mean Values in MPH Users and Matched Controls Based on 300 Posterior Draws

eReferences

This supplementary material has been provided by the authors to give readers additional information about their work.

© 2024 Garcia-Argibay M et al. *JAMA Network Open.*

eIntroduction. Bayesian Statistics

In contrast to the frequentist paradigm, which focuses on estimating population parameters from observed data, Bayesian statistics adopts a fundamentally different perspective. Rather than treating parameters as fixed but unknown quantities, Bayesians view them as uncertain entities whose credibility should be updated in light of the evidence. This update is formalized through Bayes' theorem, which mathematically combines prior information –available knowledge about the parameter– with the observed data to yield a posterior distribution. This posterior distribution reflects the degree of belief assigned to different possible values of the parameter after accounting for the data. Therefore, a Bayesian analysis revolves around constructing and interpreting the posterior distribution. This distribution encapsulates not just the most likely parameter value (mode) but also the entire range of plausible values and their associated credibility intervals. This probabilistic interpretation resonates intuitively with the clinical domain, where uncertainty and individual variability are inherent features.

The limitations of null hypothesis significance testing (NHST) are well-documented and increasingly acknowledged in medical research. Bayesian inference offers a compelling alternative, addressing these shortcomings and providing valuable advantages in analyzing clinical data. Unlike NHST's binary verdict of “significant” or “not significant,” Bayesian methods quantify the degree of evidence for both the null and alternative hypotheses. This nuanced approach avoids dichotomizing complex effects and facilitates a more informative interpretation of the data. For example, instead of simply concluding "no difference" between methylphenidate and control groups, Bayesian analysis can reveal the extent to which the data support either the absence or presence of an effect. This empowers researchers to move beyond dichotomies and make more confident and precise inferences.

Furthermore, Bayesian methods readily lend themselves to the incorporation of prior information from various sources, such as previous studies, biological plausibility, or expert knowledge. This can be particularly valuable in medical research, where data may be scarce or heterogeneous. By leveraging prior knowledge, Bayesian analyses can yield more precise and informative results than traditional frequentist methods, especially when dealing with rare events or small sample sizes.

© 2024 Garcia-Argibay M et al. *JAMA Network Open.*

eMethods. Detailed Methods

For the random intercept, a Student's *t* prior (μ=0, σ =2.5, ν=3) was used. Priors for regression slopes were flat (uniform) and the overdispersion parameter (ϕ) was assigned a weakly-informative gamma (0.01, 0.01) prior. The regression model was fitted with the log link function using 4 independent Markov chain Monte Carlo (MCMC) chains with 1000 warmup iterations and 3500 sampling iterations in each chain. Convergence was assessed by the Gelman-Rubin potential scale reduction factor ($\hat{R}$)[1] and using trace plots, and model fit was inspected using graphical posterior predictive checking procedures. Different models (Poisson, zero-inflated Poisson [ZIP], and zero-inflated negative binomial [ZINB]) were fitted and compared via leave-one-out cross validation (LOO-CV)[2] based on the expected log pointwise predictive density (ELPD). Stability was assessed using Pareto-*k* diagnostics, with values <0.7 deemed appropriate.[2] To ensure an equal rate of cardiovascular events over the baseline period, we fitted a multilevel negative binomial model comparing the 365–183 days before treatment with the 182–1 days before treatment. The overall rate of cardiovascular events was similar between 365–183 days before treatment initiation and 182–1 day before treatment (IRR=1.09, 95% HDI: 0.81–1.45).

© 2024 Garcia-Argibay M et al. *JAMA Network Open.*

eResults. Detailed Results

Model convergence was achieved in all models with a potential scale reduction ($\hat{R}$≈1.00); the trace plot suggested that the chains mixed well (eFigure 1), and all parameters had an effective sample size >9,000. Posterior predictive checking indicated that the predicted counts fitted the observed distribution of the data (eFigure 2). Model fit comparison using LOO-CV showed that the best-fitting model was a negative binomial model (see Supplementary Table S2), and all Pareto *k* estimates were less than 0.5.

© 2024 Garcia-Argibay M et al. *JAMA Network Open.*

eTable 1. Diagnoses and ICD-10 codes to define the occurrence of short-term cardiovascular events.

	Diagnosis	ICD-10 code	Primary	Secondary
Ischemic heart disease	Acute myocardial infarction	I21-I23	Yes	Yes
	Acute coronary syndrome	I21-I23 or I20.0	Yes	No
Cerebrovascular disease	Subarachnoid bleeding	I60	Yes	Yes
	Hemorrhagic stroke	I61-I62	Yes	Yes
	Ischemic stroke	I63-I64	Yes	Yes
	Other cerebrovascular disease	I65-I69	Yes	Yes
Venous thrombo-embolism	Deep vein thrombosis	I80	Yes	Yes
	Pulmonary emboli	I26	Yes	Yes
Heart failure	Heart failure	I50	Yes	Yes
Tachyarrhythmias	Atrial fibrillation/flutter	I48	Yes	Yes
	Supraventricular tachycardia	I47.1	Yes	Yes
	Ventricular tachycardia	I47.0, I47.2, I49.0, I49.8	Yes	Yes
	Cardiac arrest	I46	Yes	Yes

eTable 2. Model fit comparison among negative binomial, Poisson, Zero-inflated Poisson, and zero-inflated negative binomial.

Model	ELPD (SE)	ΔELPD (SE)
NB[1] (REF)	-464.1 (17.4)	
POI[2]	-479.6 (20.2)	-15.5 (5.3)
ZIP[2]	-472.6 (18.7)	-8.5 (3.1)
ZIPNB[2]	-466.6 (17.8)	-2.5 (1.7)

Note. Model comparison using leave-one-out cross validation (LOO). ELPD = log pointwise predictive density.

[1]All Pareto k estimates are good ($k < 0.5$).

[2]All Pareto k estimates are ok ($k < 0.7$).

© 2024 Garcia-Argibay M et al. *JAMA Network Open.*

eFigure 1. Trace plot of the four MCMC chains after burn-in.

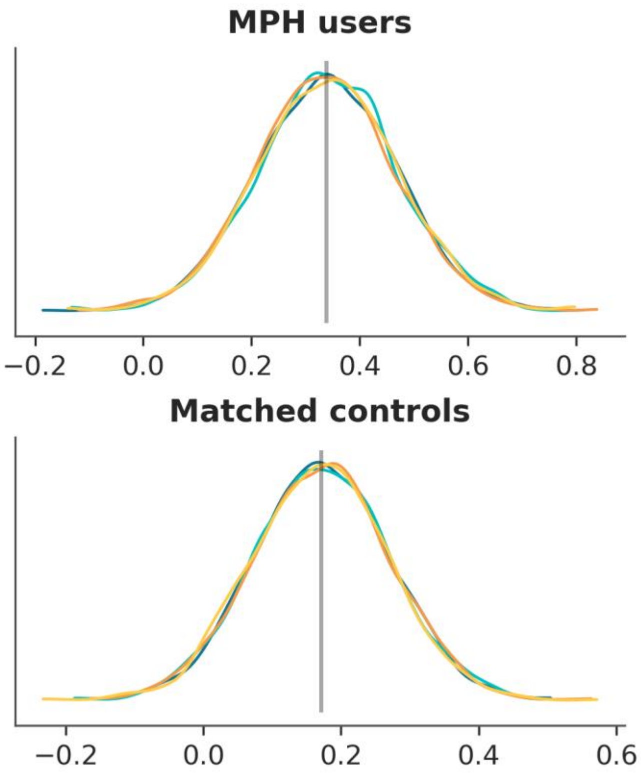

© 2024 Garcia-Argibay M et al. *JAMA Network Open.*

eFigure 2. Graphical posterior predictive checking using an overlay of density plots (left) and distribution of mean values (right) in MPH users and matched controls based on 300 posterior draws.

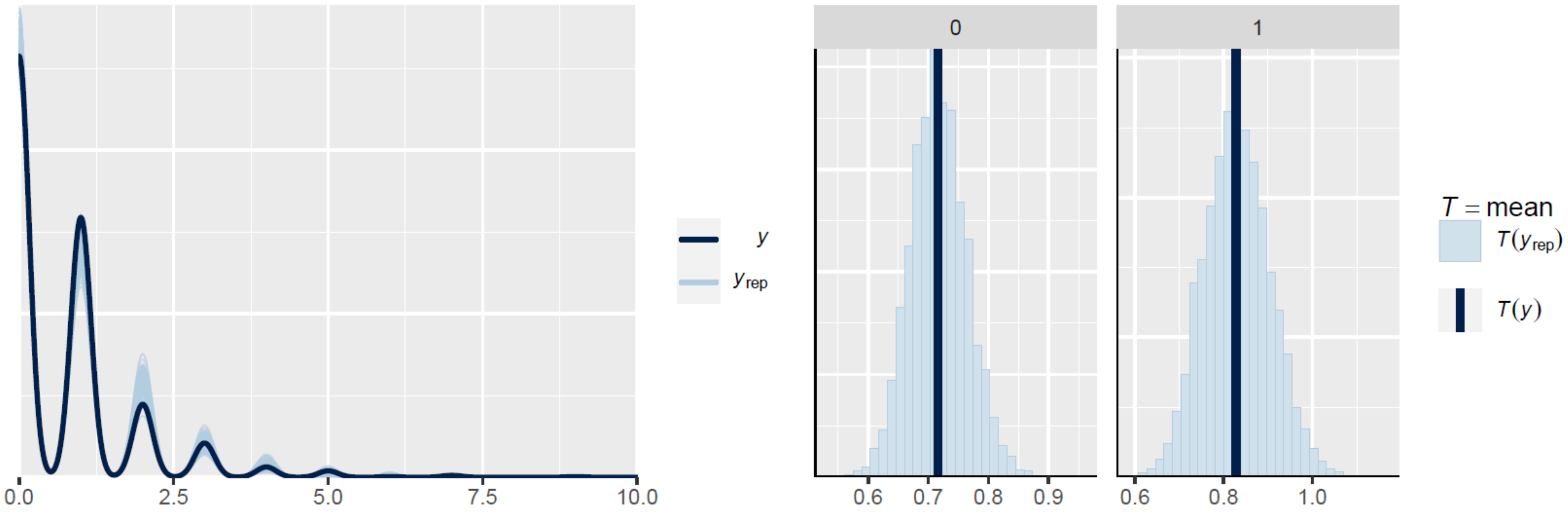

© 2024 Garcia-Argibay M et al. *JAMA Network Open.*

eReferences

1. Gelman A, Rubin DB. Inference from Iterative Simulation Using Multiple Sequences. *Statist Sci*. 1992;7(4):457-472. doi:10.1214/ss/1177011136

2. Vehtari A, Gelman A, Gabry J. Practical Bayesian model evaluation using leave-one-out cross-validation and WAIC. *Stat Comput*. 2017;27(5):1413-1432. doi:10.1007/s11222-016-9696-4

© 2024 Garcia-Argibay M et al. *JAMA Network Open.*
