# Supplementary material for: Methylphenidate and Short-Term Cardiovascular Risk
Source: JAMA Netw Open. 2024 Mar 6;7(3):e241349. doi: 10.1001/jamanetworkopen.2024.1349 (PMC10918505; doi:10.1001/jamanetworkopen.2024.1349)
Supplement: Supplement 2. — Data Sharing Statement [file jamanetwopen-e241349-s002.pdf]

Data Sharing Statement

Garcia-Argibay. Methylphenidate and Short-Term Cardiovascular Risk. *JAMA Netw Open*. Published March 06, 2024. doi:10.1001/jamanetworkopen.2024.1349

Data
Data available: No

Additional Information
Explanation for why data not available: The Public Access to Information and Secrecy Act in Sweden prohibits us from making individual level data publicly available. Researchers who are interested in replicating our work can apply for individual level data at Statistics Sweden: www.scb.se/en/services/guidance-for-researchers-and-universities/.
